# Advances in Cartilage Tissue Engineering Using Bioinks with Decellularized Cartilage and Three-Dimensional Printing

**DOI:** 10.3390/ijms24065526

**Published:** 2023-03-14

**Authors:** Roxanne N. Stone, Jonathon C. Reeck, Julia Thom Oxford

**Affiliations:** 1 Department of Mechanical and Biomedical Engineering, Boise State University, 1910 University Drive, Boise, ID 83725, USA; 2 Center of Excellence in Biomedical Research, Boise State University, 1910 University Drive, Boise, ID 83725, USA; 3 Department of Biological Sciences, Boise State University, 1910 University Drive, Boise, ID 83725, USA

**Keywords:** bioinks, bioprinting, cartilage, decellularized, extracellular matrix, tissue engineering, scaffold

## Abstract

Osteoarthritis, a chronic, debilitating, and painful disease, is one of the leading causes of disability and socioeconomic burden, with an estimated 250 million people affected worldwide. Currently, there is no cure for osteoarthritis and treatments for joint disease require improvements. To address the challenge of improving cartilage repair and regeneration, three-dimensional (3D) printing for tissue engineering purposes has been developed. In this review, emerging technologies are presented with an overview of bioprinting, cartilage structure, current treatment options, decellularization, bioinks, and recent progress in the field of decellularized extracellular matrix (dECM)–bioink composites is discussed. The optimization of tissue engineering approaches using 3D-bioprinted biological scaffolds with dECM incorporated to create novel bioinks is an innovative strategy to promote cartilage repair and regeneration. Challenges and future directions that may lead to innovative improvements to currently available treatments for cartilage regeneration are presented.

## 1. Introduction

It is estimated that more than one in five adults in the US suffer from osteoarthritis (OA) [1]. Despite the large health burden of joint diseases, clinically effective treatments to restore cartilage and new therapeutic approaches are limited [1,2,3,4]. Research into novel approaches for cartilage repair and regeneration are needed to provide new treatment options to patients. Tissue engineering approaches that combine cell therapy, the use of decellularized extracellular matrix (dECM) with three-dimensional (3D) printing methods may hold promise [1,2,4,5]. The distinction between biomaterials and bioinks for cartilage regeneration and repair as well as the behavior of cells within dECM-based bioinks for cartilage regeneration have been recently reviewed [6,7], and therefore, these topics will not be covered extensively here. However, 3D printing of structures from bioinks that include components derived from dECM for cartilage regeneration is an emerging field. This review highlights the bioprinting methods, including extrusion, inkjet, laser, and the use of a digital light printer. Additionally, the studies that are reviewed here focus on those that used a dECM source combined with a bioink to fabricate cartilage tissue. The discussion includes recommendations for future scaffold design and addresses current limitations in the field.

Scaffold manufacturing may be carried out by extrusion, inkjet, or laser-assisted bioprinting using a deposited material or bioink [2,5]. Digital light printers provide an advantage due to higher resolution without applying high stress on the cells [8]. Common to all printing methods is the use of computer-aided design based on patient-specific parameters [4,5]. The differences in bioprinting technologies are based on how each fabrication method prints the structure. OA is major challenge for both human and animals [9]. A tissue engineering approach utilizing new and novel scaffolds may promote cartilage regeneration for human and veterinary patients. To achieve this, scaffolds may incorporate specific therapeutic molecules to deliver drugs, growth factors, or extracellular molecules. Natural sources of cartilage components for the development of novel dECM-based bioinks may be derived from animal or human tissues [10,11]. A lack of information on suitable materials for recapitulating the structure and function of the cartilage ECM currently limits progress in this field. Various materials have been used for in vivo and in vitro modeling; however, currently available materials have limitations with respect to their ability to recapitulate the cartilage tissue microenvironment. The primary objective of tissue engineering is to produce a tissue that can be fully integrated into existing tissues to support normal function. Previous work has shown that scaffolds can be directly implanted into cartilage defects with positive results for up to 4 years. However, data show that implants fail over time. Activities such as walking result in fatigue stress damage to implants under cyclic loading stresses. This knowledge highlights a need for future work to explore degradation of materials over time to better predict implant fatigue and failure [12,13]. dECM-based bioinks used for cartilage repair may provide improvements to current treatment.

## 2. Methods

**Figure 1:**
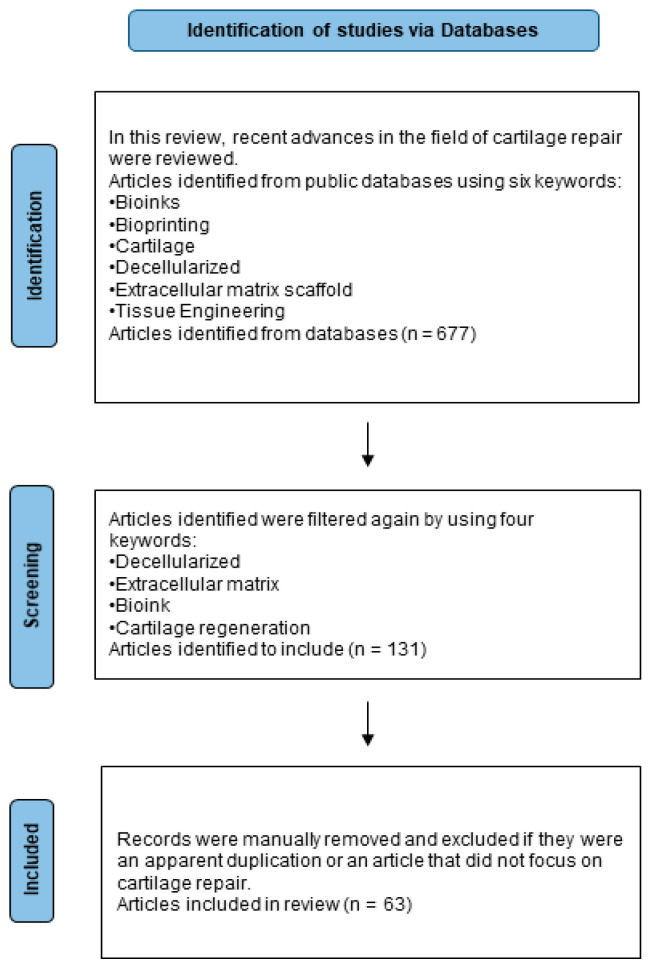
PRISMA flow chart.

The Preferred Reporting Items for Systematic reviews and Meta-Analysis (PRISMA) guidelines were used for this study [14]. Published journal articles were identified in publicly available databases through a literature search. Inclusion criteria were based on relevance to bioinks, cartilage regeneration, decellularized extracellular matrix, and 3D printing in order to identify articles relating to the development of novel scaffolds using fabrication methods that include dECM-based bioinks. The PubMed database was accessed (last date consulted 27 February 2023) A flow chart is shown in Figure 1.

## 3. Bioprinting Technologies; Advantages and Disadvantages

Extrusion-based bioprinting is the most commonly used technique for 3D bioprinting. Extrusion 3D bioprinting dispenses continuous filaments of the desired material through a nozzle. Pneumatic air pressure or mechanical force is applied to the bioink, resulting in a controlled release. The primary advantage to this method of bioprinting is its capacity to deposit high cell densities at low cost. Disadvantages are related to the print resolution of 100 µm and high incidence of cell death at higher print pressures, resulting in cell viability of 40–80%. The resolution of extrusion printers is limited by the diameter of the print nozzle (0.8–0.2 mm) and the sheer force that cells can endure. [2,4,5,13].

Inkjet bioprinting deposits droplets of the bioink in a layer-by-layer fashion. This technique uses one of three mechanisms to control the print nozzle: piezoelectric, thermal, or electrostatic forces. Inkjet bioprinting has advantages of low cost and high printing speed while maintaining high cell viabilities estimated at over 85%. However, disadvantages are that inkjet systems require low viscosity bioinks, which limits the size of the print. While small droplets allow for high resolution printing, they also lead to frequent nozzle clogging during printing [1,2,4,5,13].

Laser-based bioprinting uses a laser energy beam to deposit the bioink. An advantage to this technology is that it is a nozzle-free procedure which eliminates clogging issues. Laser 3D bioprinting has the ability to print at high resolutions, estimated at 20 µm, with the control of cell density throughout the scaffold. However, laser bioprinting has several disadvantages such as thermal damage to cells, limitation to the size of the print, high running costs, and time required to create the construct [2,4,5,13].

Digital light printers use a mechanism of photo-crosslinking hydrogels to form a layered structure or an injectable solution. A photo-crosslinked hydrogel involves a polymerizable material, a photoinitiator, and light. The UV light and photoinitiator triggers the gelation of the material at a high speed and high resolution. The desired scaffold is created through a layer-by-layer process. The most significant advantage of digital light printers is the ability to print small scaffold designs at resolutions on the order of 10 µm. A disadvantage to photo-crosslinking is that UV light can damage cell DNA, leading to reduced cell viability [8,13,15]. Each bioprinting technique has advantages and disadvantages. Limitations of each 3D bioprinting system must be considered when designing functional tissues intended for implantation. A summary of each print style is provided in Table 1.

## 4. Cartilage Properties

Cartilage is a tough, yet flexible tissue that contains large amounts of glycosaminoglycans (GAGs), such as chondroitin sulfate and hyaluronic acid (HA), electrostatically bound to type II, IX, and XI heterotypic collagen fibers. Proteoglycans, such as aggrecan, are the predominant molecular constituents of articular cartilage [1,3,16,17,18]. Collagen fibrils are oriented parallel to the articular surface within the superficial zone.

**Figure 2:**
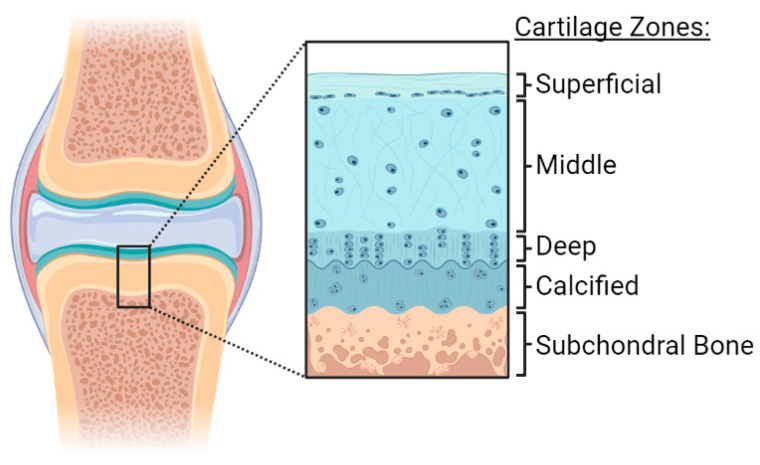
Articular cartilage organization. The structure of the articular cartilage is organized into three distinct zones: the superficial, middle, and the deep zones. Calcified cartilage lies between the subchondral bone and the deep zone. Created with BioRender.com.

The precisely organized architecture of cells and the ECM provides the tissue’s normal structural integrity. The structure of the articular cartilage is organized into three distinct zones, the superficial, middle, and the deep zones, as illustrated in Figure 2. The superficial zone is a thin layer composed of collagen fibers, low GAG content, and chondrocytes. The superficial zone protects the deeper zones from tensile and shear stress. The thicker middle zone has a relatively low number of cells and is composed primarily of collagen and aggrecan. The deep zone contains a high content of GAGs with collagen fibrils aligned with the chondrocytes, which are organized in a columnar fashion. The calcified cartilage lies between the subchondral bone and the deep layer and represents the mineralization junction that anchors the collagen fibrils of cartilage to the subchondral bone [2,3].

The deep zone provides the highest resistance to compressive forces. This chondrocyte organization, collagen orientation, and GAG content provides a Young’s modulus of cartilage between 0.2 and 2 MPa [2,3,19]. The primary function of the articular cartilage is to protect the subchondral bone from mechanical forces by distributing the load equally while maintaining low friction across the surfaces [1,16,17,18]. Collagen fibrils are oriented perpendicular to the articular surface and serve as the fibers in this fiber reinforced composite material resisting compressive forces [2,3,19]. Articular cartilage degeneration begins at the surface of the synovial joint characterized by the onset of fibrillation, disrupting the smooth surface of the articular cartilage. Fibrillation is triggered by mechanical forces that damage the tissue. As the damage progresses at the tissue level, molecular changes also occur. Collagen fibrils beneath the articular surface may be disorganized and the tissue may lose HA and aggrecan content [17,18]. As the damage progresses at the tissue level, molecular changes also occur. A better understanding of the cartilage degeneration mechanism is required to develop new treatments to repair damaged cartilage.

## 5. Current Treatments

OA is a chronic, debilitating, and painful disease and one of the leading causes of disability and socioeconomic burden [1,3,17,18,20,21]. It is estimated that 250 million people are affected worldwide [2]. Currently, there is no cure for OA. Treatment options depend on the stage of OA and are palliative to alleviate chronic pain and delay surgical intervention [1,2,3,17]. In early stages the recommended treatments focus on alleviating the symptoms of chronic pain using topical or oral medications. In more advanced stages, intra-articular injections of corticosteroids are recommended [2,17,22].

Cell-based therapies have shown promising results in treating cartilage defects. Autologous chondrocyte implantation focuses on promoting regeneration by using the implantation of autologous chondrocytes into the joint [2,23]. Another approach is that of matrix-induced autologous chondrocyte implantation, which uses harvested autologous chondrocytes on porcine-derived type I and II collagen matrices. The matrices are able to stimulate and direct cell growth before implantation [4,23].

Other approaches focus on bioactive scaffolds to recruit native endogenous cells. One promising example is the use of particulated allograft cartilage ECM. As a bioactive scaffold, this material may be placed in a defect and mixed with platelet-rich plasma or bone marrow concentrate and secured in place using a fibrin glue sealant. Commins and colleagues determined that a bioactive cartilage ECM scaffold could improve cell adhesion and migration and provide interleukin-1 receptor antagonist protein to the wound site using equine mesenchymal stem cell (MSCs) and chondrocytes. Proteomic analysis of the scaffold confirmed the presence of bioactive proteins involved in cartilage homeostasis [24]. Based on this approach, biomaterials could be designed to promote cell adhesion and differentiation and be deliverable in a minimally invasive manner for cartilage lesions and OA treatment [2,17,18,24].

Cell-based approaches may have advantages over other strategies as cells have the capability to remodel the ECM and secrete soluble factors such as cytokines and growth factors. Stem cells, undifferentiated and unspecialized cells that can self-renew and give rise to one or more specialized cell [5,25,26,27], can contribute to ECM formation and perform processes that are essential for creating a functional tissue [1,25]. MSCs have the capacity to differentiate into chondroblasts, which further differentiate into chondrocytes, which produce the ECM of cartilage. The multipotential capacity of MSCs has enabled clinicians to use genetic engineering approaches to manipulate the cells and grow cartilage in osteochondral defects. Studies have shown the successful generation of a layer of hyaline cartilage [2,28].

Cells for tissue engineering may be collected from several suitable sources, including bone marrow, peripheral blood, adipose tissue, muscle, dermis, synovium, umbilical cord blood, placenta, and dental tissues. Challenges include the clinical transformation into the desired tissue, quality control, and production strategies that yield a functional tissue that is economically viable [17,18,25,27,29,30,31]. Bone-marrow-derived MSCs have been used in clinical trials for articular cartilage repair. Adipose-derived stromal cells (ADSCs) are an attractive alternative and can be harvested in a manner that does not negatively impact patient mobility at the donor site. Both MSCs and ADSCs have demonstrated potential for differentiation into chondrogenic cells for cartilage tissue engineering and expression of chondrogenic transcription factors [1,2,18,26,32]. Recent studies have shown that the combined use of both chondrocytes and MSCs enhanced chondrogenesis. Levato and colleagues used a multipotent approach by combing articular-cartilage-resident chondroprogenitor cells from the superficial zone with MSCs and chondrocytes to create bioprinted cartilage constructs using cells specific for the different zones [33]. Induced pluripotent stem cells (iPSCs) have been used to create hyaline-like cartilage [2,5]. iPSCs can be derived from the patient and therefore have the potential to differentiate into adult cells that are patient-specific [3]. Personalized reprogramming of the cells and the use of specific biomaterials to deliver cells and signaling factors to the defect area may lead to improved outcomes in tissue regeneration [1,2,5,17,18].

## 6. Decellularization as a Strategy to Create Cartilage Biomaterial Constructs

Scaffolds with dECM are currently being investigated for use in the treatment of osteochondral defects and for the regeneration of cartilage [1,3,34,35,36,37,38,39]. While decellularization techniques have been developed and used to create a biological ECM from cells in culture, tissues, and organs, it is possible that material within the scaffold could have an adverse effect if not removed. For example, residual cellular and genetic material may induce adverse immune response and rejection [26,30]. Multiple approaches and techniques have been developed to prepare dECM from cartilage tissue. The goal of decellularization methods is to remove cells and DNA from the tissue while preserving ECM structural components and mechanical integrity [30,35,40]. The resulting scaffold may modulate the environment that the cells experience to promote cell proliferation and differentiation essential for repair and regeneration [26,34,35]. Sodium dodecyl sulfate (SDS) treatment has been shown to remove at least 90% of the cellular DNA. Treatment with 2% SDS for 8 h resulted in the greatest decrease in DNA [16,41]. Ethanol can be used to defat samples and guanidine hydrochloride, and sodium acetate can be used to denature and remove noncollagenous components [42]. NaOH may be used to remove cells and increase the porosity, while NaCl can disrupt ionic interactions, removing some proteins, while retaining essential growth factors with minimal damage to the ECM architecture [26,42,43]. Freeze-thaw cycles may help to increase the porosity associated with ice crystal formation [30,40,41,43,44,45]. Lyophilization has been shown to help with cell disruption and removal of cellular components [26,46,47].

**Figure 3:**
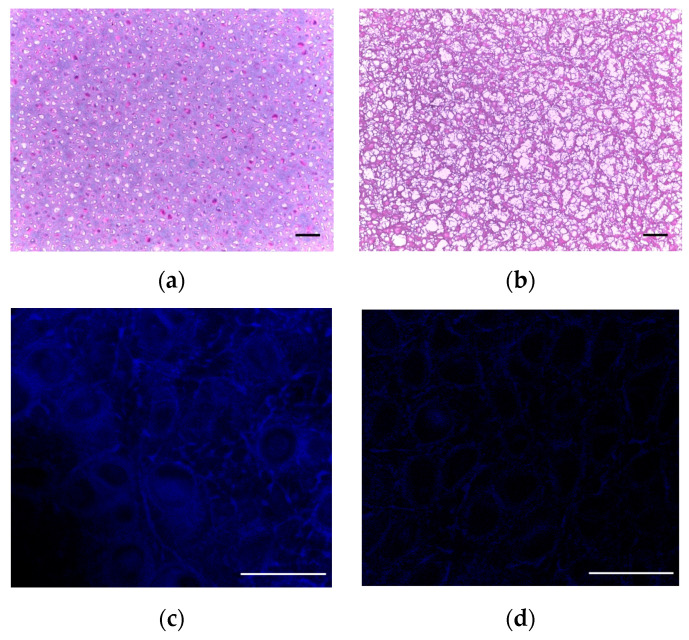
Visualization of cartilage tissue by histology. (**a**) Nondecellularized porcine cartilage stained with H&E and (**b**) H&E stain on final decellularized cartilage, transverse image. (**c**) Hoechst stain to fluorescently visualize DNA (blue). Arrow indicates nucleus of a cell. (**d**) Hoechst stain shows absence of DNA after decellularization process (see absence of blue). Scale bar = 100 µm for (**a**,**b**); scale bar = 50 µm for (**c**,**d**). Adapted with permission from Ref. [49] 2021, Stone and Oxford.

**Figure 4:**
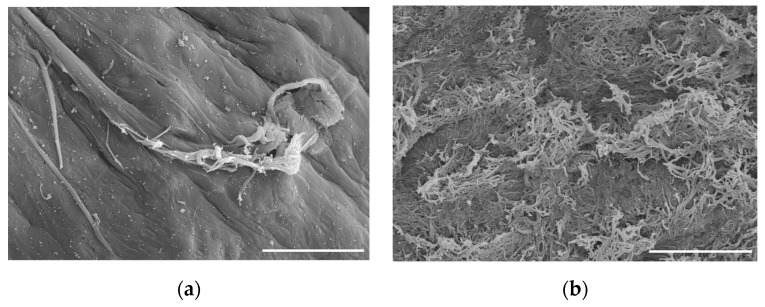
Visualization of cartilage by scanning electron microscopy. (**a**) Cartilage prior to decellularization. Tissue appears visibly smooth and intact. (**b**) Final decellularized cartilage surface. Tissue displays increased surface area and exposed collagen network. Scale bar = 20 µm. Adapted with permission from Ref. [49] 2021, Stone and Oxford.

Deoxyribonuclease and ribonuclease are commonly used to remove DNA and RNA during repeated treatments [16,30,35,40,43]. Removal of 99% of genomic information has been observed after a 6-day wash cycle [43]. Since residual DNA is directly correlated with implant rejections, a benchmark of <50 ng DNA per mg of dECM dry weight and <200 base pair DNA fragment length has been established [3,26]. Histological examination of the resulting decellularized scaffold has been used to evaluate samples for the absence or presence of residual cells using hematoxylin and eosin staining, as shown in Figure 3 [16,40,42,43,48,49]. Proteoglycans and GAGs can be assessed using Safranin O staining [16,40,48]. Scanning electron microscopy may be used to visualize the porosity and to assess the structure before and after the decellularization process as shown in Figure 4 [40,42,48,49].

Mass spectrometry can be used to assess the protein content of the scaffold before and after decellularization. Recent results indicate that while many of the structural proteins remain after the decellularization process, soluble, non-crosslinked components are removed from the tissue [49]. Additionally, proteomics can be used to monitor the response of MSCs to the decellularized tissue scaffold. Limited proteomic studies of tissue-engineered cartilage are available, and additional information would be helpful [50]. Differential proteomic analysis can also improve our understanding of cell-biomaterial interactions [26,49]. Biomechanical characteristics of the decellularized scaffold may be used to determine the tissue strength, stress, and strain. Load displacement curves can be used to determine tissue strength, which is important for cartilage as a load-bearing tissue [30,40,41,42,43,48].

**Figure 5:**
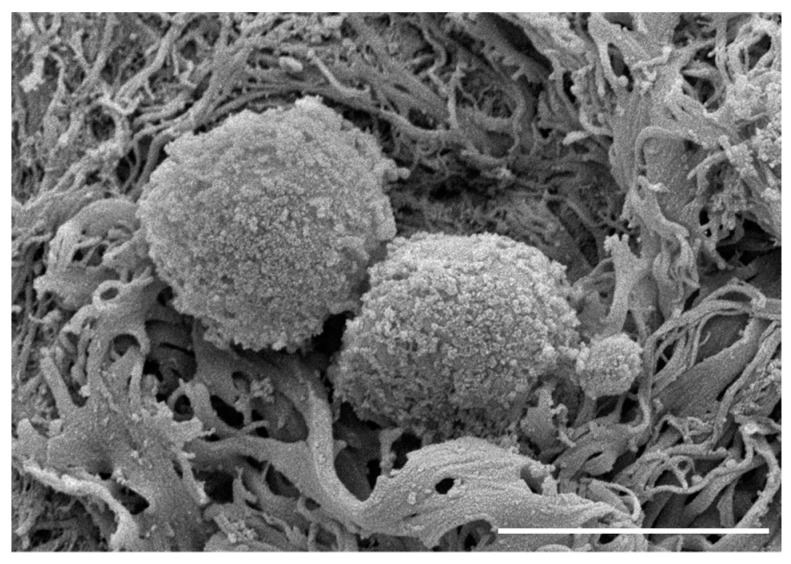
Visualization of cartilage with C28/I2 chondrocytes on decellularized scaffold by scanning electron microscopy. Cells attached to scaffold after 1 week in culture. Scale bar = 20 µm.

Scaffolds composed of dECM may be sterilized as the final step before the recellularization process begins [26,46,47]. Recellularization of dECM scaffolds requires that cells have access to the internal compartments of the scaffold. Dense dECM may hinder the recellularization of the scaffolds, causing the cells to proliferate on the surface in comparison to more central locations of the scaffold, as shown in Figure 5. The formation of physical channels may allow for sufficient recellularization [34,43,49].

While there currently is no standard decellularization method, chemical, physical, or combinative methods are usually employed. Detergents and physical methods of disruption have been shown to damage collagens and disrupt the ECM, reducing the strength and mechanical properties of the ECM [10,35]. A combination of dECM with 3D bioprinting technologies would allow the inclusion of proteins of the natural ECM of cartilage that contains components that are essential for cell adhesion, growth, and differentiation [3,10,34,35,51].

Replicating the tissue-specific composition of the ECM may provide inherent biochemical and biophysical cues that can promote cellular function and development that biomaterials alone lack. Additionally, addition of dECM to a bioink must take into consideration printability, cell compatibility, mechanical properties, and the capacity for remodeling. Printability is determined by the bioprinter configurations and dependent on the properties of the bioink. Bioinks with dECM must have an appropriate viscosity for the intended 3D bioprinting technology, which is determined by the nature of the dECM the molecular weight of individual constituents, the structure, and interactions between the components [13]. Sourcing tissues from human donors requires invasive harvesting procedures and there are moral and ethical concerns related to cadaver donors. Low availability is also an issue with human dECM tissues. A recent study by Ayariga and colleagues explored the decellularized avian cartilage as an alternative to bovine and porcine tissues [11]. In addition, variables such as the donor ECM age should be considered, as age may influence the dECM properties. Comparative studies on young and old tissues indicate that pore sizes and ECM thickness vary significantly in young verses old articular cartilage. Scaffolds produced from older tissue have lower porosity, which may interfere with cell migration and scaffold degradation rates [10,11,35]. Additional research is needed to address questions regarding the design and production of decellularized constructs to minimize biosafety concerns and maximize clinical applicability. Needed studies include comparing different dECM materials for incorporation into bioinks and their compatibility with existing 3D bioprinting technologies [11,13].

## 7. dECM and Bioinks for Cartilage Regeneration and Repair

Fabrication of the dECM for supplementing standard bioinks may improve decellularized scaffolds effectiveness [34,35]. Bioprinting and biofabrication techniques may produce products that are biologically functional by incorporating cells at defined coordinates within a 3D matrix prepared in a layer-by-layer fashion. Materials for bioinks used in bioprinting and biofabrication must support high cell viability, facilitate cell proliferation, and regulate cell differentiation [1,4,19,31,52]. Due to the inherent differences in microenvironments when transitioning from cartilage to bone, 3D bioprinted scaffolds may provide an approach that would allow the recapitulation of the intricate structure of the joint surface and provide an authentic construct that would support cartilage regeneration within a lesion [4,31]. To stimulate regeneration of an osteochondral defect, the scaffold must induce regeneration of both bone and cartilage. To promote osteochondral tissue regeneration, the scaffold must modulate cellular processes of multiple cell types [31]. A limitation to biofabrication of cartilage scaffolds is the load bearing capacity of the resulting tissue and intermediate scaffold, which must bear the loads generated through daily movement and exercise. Enhancement of the printability and mechanical stability of dECM-based bioinks may be accomplished by addition of a polymer or the introduction of crosslinks [3,18,53].

Bioinks available for tissue engineering include agarose, alginate (Alg), cellulose, collagens, gelatin, HA, poly (ethylene glycol) diacrylate (PEGDA), and silk. Agarose is biocompatible, shows the ability to support chondrogenesis, and has a stiffness and viscoelasticity comparable to native cartilage. However, in clinical trials, agarose degrades and demonstrates bio-inertness limitations [1,5]. Alg is a readily available natural polymer widely used in cartilage bioprinting. Alg is a hydrophilic, negatively charged polysaccharide that crosslinks in an easily fabricated manner. It does not elicit an inflammatory response when implanted and therefore has excellent biocompatibility. A limitation to Alg is that it is bioinert and has limited degradation; therefore, to maintain high resolution and structural integrity, Alg is often printed in conjunction with other materials [1,2,4,5]. Cellulose has been considered for cartilage bioprinting, and 3D bioprinting of nanofibrillated cellulose with alginate has also been studied for its ability to recreate cartilage tissue. This type of bioink is beneficial because it improves flow properties leading to the formation of complex geometries with high resolution [1,5].

Collagen, the most prevalent protein in articular cartilage, is a common component for scaffolds used in cartilage tissue engineering. Collagen-based hydrogels have biocompatibility and advantageous biodegradation properties. Modifications using cross-linking have shown improved mechanical strength [1,5]. Gelatin is partially hydrolyzed collagen that has favorable biological properties. Gelatins are characterized by cell adhesion properties, immunogenicity and low antigenicity. Modification with cross-links to form hybrid materials increases its stability. Gelatin methacrylate (GelMA) is used in cartilage tissue engineering due to resemblance to ECM, which helps promote cell adhesion and proliferation, as well as its rheological properties and high compressive modulus [1,5]. HA is abundant in articular cartilage and therefore a logical material choice for fabrication of tissue engineering scaffolds for cartilage formation. HA hydrogels are currently used for chondral restoration, and HA scaffolds support cell attachment and induce chondrogenesis [1,5]. PEGDA hydrogels possess biocompatibility and the capability to be loaded with specific cells and biological factors. UV cross-linking has been shown to improve mechanical properties [5,8,54]. Silk fibroin (SF) is a protein polymer that is biocompatible, cytocompatible, possesses mechanical strength, and is biodegradable. SF is derived from the silkworm cocoon and has characteristics that may be advantageous to cartilage tissue engineering [5,19].

## 8. Recent Progress

Wu and colleagues investigated a bioink composed of porcine articular dECM, infrapatellar fat pad adipose–derived stem cells from the knee joints of New Zealand white rabbits and sodium alginate. Their study demonstrated that the compression modulus increased in proportion to the content of dECM and that higher concentrations of dECM supported cell aggregation and SOX-9 expression. These results suggest that dECM can facilitate differentiation down the chondrocyte lineage [55]. Photo-crosslinking of scapular dECM with photo-responsive HA was investigated in combination with chondrocytes derived from rabbit auricular cartilage to form an injectable hydrogel by Xu and colleagues. After 8 weeks of subcutaneous implantation in nude mice, mature cartilage tissue formed, indicating that the dECM provided features of the cell’s native microenvironment and was suitable for filling and repairing defects. Quantitative analysis in this study showed that after four weeks the cell-hydrogel construct no longer increased in DNA content, but had continued increase in collagen and GAG content [22]. Tissue specific dECM hydrogels as bioinks used for 3D bioprinting and or in the form of an injectable are showing promise for cartilage tissue engineering applications.

**Figure 6:**
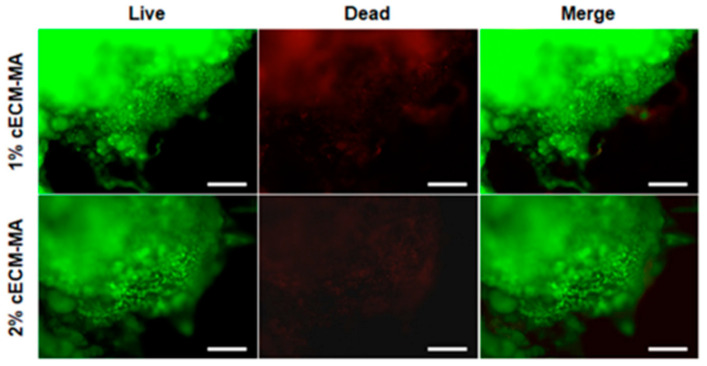
Cell viability 7 days following encapsulation in cECM-MA. Living cells are stained in green, and dead cells are stained in red (scale bars = 200 µm). Adapted under open access Creative Commons CCBY 4.0 license from Ref [56].

Behan and colleagues developed a methacrylated cartilage ECM-based hydrogel/bio-ink using photocrosslinking and goat bone marrow-derived MSCs. This study evaluated varying percentages of porcine articular cartilage dECM and gelatin with and without methacrylation. Results indicated that higher dECM concentrations may delay full crosslinking of available sites within the ink. The presence of cells had no significant effect on the bioinks’ behavior. It was concluded that concentration of the dECM in formulations have measurable effects on rheological properties. The inks supported high cell viability and chondrogenesis, as shown in Figure 6 [56].

Visscher and colleagues investigated a photo-crosslinkable dECM-based bioink for patient-specific cartilage constructs. Methacrylate was used to chemically modify the porcine ear dECM to provide rapid structural integrity after the printing process. Rabbit auricular chondrocytes were mixed into the bioink formulations containing different concentrations of dECM. Results indicated that stiffness of hydrogels significantly increased with increasing dECM concentrations. Increased dECM concentrations lead to increased cell proliferation and ECM production and controlled the morphological phenotype of the chondrocytes on the scaffold. Bioinks may provide constructs that are effective therapeutic approaches for personalized cartilage reconstruction [51]. Methacrylated dECM was used in a study of the biomechanics and bioactivity of tissue constructs carried out by Beck and colleagues. Rat bone marrow derived MSCs were encapsulated in scaffolds containing 10% and 20% porcine knee dECM. Gels with dECM showed an increase in collagen type II, SOX-9, and aggrecan. The concentration of dECM was found to affect chondroinduction and mechanical properties, with 20% dECM gels possessing superior mechanical properties and promoting ECM synthesis. However, a 10% dECM gel was found to be superior in chondroinduction [53].

Improvements to dECM bioink mechanical stability was also explored by Zhang and coworkers by adding SF as a component. Results suggested that the physical crosslinking between SF and goat articular dECM in the bioink provided improved mechanical properties and a lower degradation rate when compared to the SF-only bioink. The compressive modulus of the SF-dECM bioink was within the range of soft tissue regeneration but was lower than that of native human articular cartilage. This study also evaluated the use of incorporating growth factors into the constructs to promote chondrogenic differentiation in rabbit bone marrow–derived MSCs. Results suggested that TGF-β3 under controlled-release conditions increased collagen content in the constructs. These scaffolds were further evaluated to promote cartilage regeneration by using an in vivo nude mouse model. After 28 days, cartilage lacunas and chondrocyte-like cells were observed [19]. In another study, fibrin glue hydrogel scaffolds were combined with swine neonatal meniscal cells and three different concentrations of endostatin to assess the impact on the chondrogenic phenotype. Endostatin is involved with cell differentiation during cartilage development and favors the accumulation of ECM. Investigators were able to detect markers of cartilaginous-like tissue and early markers for chondrogenicity at higher concentrations [57].

Work from Herrera Millar and colleagues investigated the role of vascularization in osteochondral repair. Endothelial cells within the vessels of meniscus produce endostatin which is important for the development and maintenance of avascular zones. The meniscus has non-vascularized regions adjacent to confined blood vessels. Using biofabrication, spatial distribution of vascular components can be designed into the scaffold. The porosity created by the interconnected network can lead to capillary ingrowth into non-vascularized tissue to yield better matrix integrations beneficial for osteogenesis and bone regeneration [26,57,58,59]. Terpstra and colleagues investigated endothelial-cell-laden and anti-angiogenic bioinks. The scaffold was design from bioactive matrix-derived microfibers and mixed individually with type I and II collagen sponges and equine stifle articular cartilage dECM with varying microfiber geometries. The results of this study indicated that a vessel network may be incorporated into a bioprinted construct, supporting the establishment of the different zones in osteochondral tissue. Biofabrication of structures with a boundary between avascular and vascularized tissue interfaces are design features that can influence the regenerative process [59].

Eicholz and coworkers investigated how scaffold microarchitecture influences the healing of large bone defects. Scaffolds were fabricated to have a large fiber diameter with low porosity and smaller fiber diameter with a higher porosity. Both scaffolds were designed to have similar surface areas. Previous research has shown that larger pore size, surface area, and good interconnectivity favored enhanced vascularization and bone formation in vivo. The results of this study indicate that scaffold architecture should be carefully controlled to regulate vascular ingrowth and osseointegration [58]. Qin and colleagues investigated a bilayered co-culture scaffold that accommodated the differentiation requirements of cartilage and subchondral bone. The scaffolds were constructed with rabbit chondrocytes in an upper gellan gum hydrogel and human placental MSCs in the lower part of the scaffold composed of a bioceramic containing gellan gum hydrogel. The bioceramic particles incorporated into the scaffold released ions to build a bioactive environment stimulating the cells in the scaffold towards specific differentiation. The scaffolds were further explored in their ability to repair by being implanted into osteochondral defects in rabbits for 12 weeks [31].

In another study, Yuan and colleagues incorporated magnesium into a swine articular dECM scaffold for cartilage regeneration. Magnesium is beneficial to skeletal growth and development and when deficient in the body is linked to cartilage lesion development. This research explored magnesium as a therapeutic ion for stimulating cartilage and bone repair in a dose dependent manner. Results from this study showed scaffolds with magnesium promoted cell proliferation and chondrogenic differentiation of rabbit bone marrow MSCs [60]. Han and coworkers investigated the incorporation of particles using electro-writing techniques for cartilage regeneration. Methacrylated HA and PEGDA scaffolds were mixed with porcine knee dECM in specific ratios and seeded with rabbit bone marrow MSCs cells. Experimental groups also contained TGF-β1 and BMP-7 in the upper layers of the scaffold, with TGF-β1 and IGF-1 in the lower layers. The addition of TGF-β1 and BMP-7 showed improved chondrogenic activity. IGF-1 with TGF-β1 enhanced the production of type II collagen. TGF-β1 is a key growth factor for cartilage differentiation and can promote cartilage formation, and may be essential for maintaining cartilage differentiation in vitro. While the results from this study showed promotion of cellular activity, the mechanical properties of the scaffolds still needed to be improved [54].

Bedell and colleagues investigated the relationship between biomaterials, cells, and other factors within osteochondral tissue. This approach used two bioinks to evaluate the effects of gelMa, β-tricalcium phosphate, and methacrylated hyaluronic acid to support growth and differentiation of human bone marrow MSCs. Incorporation of 10% *w*/*v* β-tricalcium phosphate increased the cell mineralization response within a mixed osteogenic and chondrogenic culture medium environment. This study conclude that future studies need to be conducted to understand the effect of printability additives and the bioinks’ crosslinked mechanical properties [61]. Rathan and coworkers reinforced the matrix of a scaffold with stiffer polymers to improve the mechanical properties. This study developed a 0.2% and 0.4% (*w*/*v*) porcine knee dECM and alginate bioink with human bone marrow–derived MSCs. The chondroinductive potential and capacity to act as a TGF-β3 delivery system was also investigated. The use of polycaprolactone (PCL) reinforced the bioinks framework and resulted in a compressive modulus of 0.33 MPa, which is within range of native articular cartilage. No noticeable loss in cell viability was observed with PCL reinforcement. Furthermore, the bioinks used to deliver TGF-β3 were found to promote a more robust chondrogenesis of MSCs [48]. Investigators also developed a preclinical animal model using swine bone marrow stem cells and evaluated the levels of *SOX-9, Col2a1*, and *ACAN* gene expression. The bioinks showed similar results despite the differences in species cell type [62].

In another study, investigators reinforced the scaffold matrices to improve mechanical properties by using electrospinning; Chen and coworkers dispersed electrospun gelatin and poly lactic-co-glycolic acid fibers in a bovine scapular dECM based bioink at different percentages. Scaffolds were seeded with rabbit chondrocytes and implanted into nude mice for 4 and 8weeks along with New Zealand white rabbits for 12 weeks. Results indicated that the fibers improved stiffness and toughness of the scaffolds. The electrospinning fibers also caused the strands of the scaffold to appear more fibrous and porous. It was concluded that the highest fiber percentage, 50%, demonstrated the highest type II collagen formed, indicating suitability for regeneration and remodeling of cartilage defects [63]. Electrospun PCL nanofibrils were fabricated and coated with bovine articular dECM to mimic the microenvironment of cartilage structurally and biochemically in a study carried out by the laboratory of Kim. Kim and coworkers used human ADSCs without additional biological growth factors to induce chondrogenesis. The dECM decorated nanofibrils exhibited the highest expression of chondrogenic markers such as SOX-9, collagen type II, and aggrecan. These scaffolds performed well after being implanted into an osteochondral defect of a rat model for 12 weeks [32].

## 9. Future Directions and Concluding Remarks

Despite noteworthy advances in tissue engineering technologies, several challenges remain. To successfully engineer cartilage-like tissue, continued research to develop and study biomaterials that are capable of regenerating microenvironments present in diverse tissues such as cartilage is needed. The biomaterials also need to possess the capacity to deliver growth factors and other regulatory biomolecules and maintain the mechanical integrity. By using the combination of biomaterials and biofabrication strategies, a laboratory generated implant scaffold for cartilage repair could be achieved as an alternative treatment option for osteoarthritis. There is clearly a need for novel biomaterials capable of cell and growth factor delivery that can be used in cartilage matrix regeneration. More research is needed to understand ECM proteins and their individual influence on cartilage structure and repair. Decellularization strategies vary for different tissues, and future work must investigate how decellularization methods impact the final tissue. Studies must also address the role of magnesium on the microarchitectural and physicochemical properties, cytocompatibility, and the ability of dECM scaffolds to enhance chondrogenic differentiation of cells.

Good manufacturing processes are needed to extract, purify, expand, bioprint, and differentiate stem cells into the desired tissues [27]. Additionally, veterinary medicine and human medicine can work together to address the common challenge of osteoarthritis and cartilage repair and regeneration. Veterinary medical research and medical research focusing on human disease can work together to better understand different forces such as compression, traction, and sliding on implants. Investigations addressing animal sources of tissues for human tissue engineering purposes require the establishment of donor criteria recommendations for parameters such as age, tissue thickness, porosity, and species of the source material.

Despite attempts to use a single cell line to regenerate cartilage, recently the combination of multiple cell lines, such as MSC and chondrocytes, demonstrated enhanced chondrogenesis. Research is needed to better understand cell-to-cell and cell-to-biomaterial interactions. Previous studies have shown that dECM bioinks provide superior microenvironments; however, the degradation rate presents challenges. The addition of dECM can improve adhesion properties of cells to materials. Studies reviewed here have confirmed that dECM can play a role in promoting cell activity by changing the cellular microenvironment, which regulates the behavior of cells, cell proliferation, differentiation, and cell attachment. Investigations into the role of dECM components on the self-assembly and organization of the newly synthesized ECM by resident cells may increase our knowledge about how to fine-tune the dECM used. Fatigue testing is needed to understand the mechanical properties of resulting constructs and to predict the effects of human physiological activity on implant designs. The shape of biomaterials is predicted to change under different types of loading based on how stress is distributed. More in vivo testing needs to be done on scaffolds with different percentages of dECM and porosities to provide demonstration of how real-life events affect degradation rates of different scaffold designs. Degradation is one of the reasons for implant failure; however, our current understanding of how the rate of degradation and the tissue microenvironment in combination with physiological loads determines the outcome of the implant is limited [11,12,13].

Future work investigating the formation of blood vessel networks is needed to create functional tissues. This comprehensive strategy may promote cell maturity, cell signaling, and vascularity needed to trigger tissue specific differentiation processes capable of recreating cartilage layers. The boundary between avascular and vascularized tissue, such as in cartilage and bone, is a challenge when integrating a scaffold for repair. Tissue interfaces may be developed by creating a multiphasic construct using growth factors such as BMP and TGF-β to simultaneously differentiate cells towards osteogenic and chondrogenic lineages. When designing 3D scaffolds, it is important to understand how the microarchitecture influences behavior of cells. Traditional 3D printed scaffolds range from smaller fiber diameters of 20 µm to larger fiber diameters of 200 µm. There are limited studies of materials for smaller filaments. The fiber spacing and pore size, ranges from 100 mm to 300 µm. This range of porosity has been shown to optimize cell density and ECM synthesis. In some studies, the bioinks failed to form stable filaments by bridging gaps above 1 mm. Dimensions of scaffolds in the x, y, and z dimensions are restricted by the 3D printer print-well sizes [9,58,59].

Parameters to optimize fabrication of a porous framework are needed. A customizable framework of interconnected pores as well as a large surface area may be beneficial for cell attachment, proliferation, cell communication, along with gas and nutrient exchanges required for tissue regeneration. Permeability is an essential parameter to consider when designing new scaffolds to meet biological requirements. A recent study by Prakoso and colleagues discussed tortuosity on scaffold design. Scaffolds with tortuous architectures have been shown to provide better cell attachment in comparison to scaffold designs with straight microchannels due to the increased surface-to-volume ratio and transit of essential molecules through the microchannels. Additional investigation on how porosity determines the characteristics and properties of tissue engineered scaffolds is needed [64]. Bioprinting outcomes desired for certain applications can be better understood by studying printing conditions under controlled pressure, speed, and light exposure. Denser hydrogels are required for optimal extrusion characteristics; however, softer hydrogels support biological processes such as cell proliferation, differentiation, and ECM production. The fabrication and cell culture windows need to be balanced when developing bioinks with both ideal properties.

The use of cartilage dECM in 3D bioprinting is attractive because it may provide specific cues to the cells as they undergo chondrogenesis. Characteristics such as surface topography and fiber organization can be influenced by dECM when incorporated under specific geometries to induce zone-specific tissue architecture. dECM-functionalized bio-inks are found to enhance chondrogenesis in a concentration dependent manner; however, there is a need to better understand the effects of dECM at different concentrations on the expression of key biomarkers. Key cartilage markers for these evaluations are collagen type I and II, aggrecan, SOX9, and VCAN. Sox9 is a transcription factor that plays a key role in early to mid-stage differentiation phases of chondrogenesis. VCAN is known for its role in regulating the microenvironment that supports hyaline cartilage formation by interacting with cells and other ECM molecules [9,60,61]. In addition to cartilage formation, studies have shown that the presence of dECM may also reduce the inflammatory response at the injury site, which may enhance healing [11].

In conclusion, continued efforts are required to better understand the potential of incorporating dECM into hydrogels and 3D bioprinting due to the advantages that dECM may provide for cartilage repair for joint disease and OA. A tissue engineering approach utilizing new and novel scaffolds may support cartilage repair and regeneration for human and veterinary patients.

## Figures and Tables

**Table 1:** Summary of Bioprinting Technologies *.

Technology	Extrusion	Inkjet	Laser	Digital Light
**Estimated Print Cost**	Low	Low	High	Medium
**Estimated Print Speed**	Medium Range of 10 µm–50 mm/s	Fast Range of 1–250,000 droplets/s	Fast Range 200–1600 mm/s	Medium-Fast 0.5–15 mm/s
**Resolution**	100 µm	10 µm	20 µm	10 µm
**Cell Viability**	40–80%	85%	95%	85%

* [2,4,5,8,13,15].

## Data Availability

Data sharing is not applicable to this article as no new data were created or analyzed in this study.

## Abbreviations

ADSCs	Adipose-derived stromal cells
Alg	Alginate
dECM	Decellularized ECM
ECM	Extracellular matrix
GAGs	Glycosaminoglycans
GelMA	Gelatin methacrylate
HA	Hyaluronic acid
iPSCs	Induced pluripotent stem cells
MSCs	Mesenchymal stem cells
OA	Osteoarthritis
PCL	Polycaprolactone
PEGDA	Poly(ethylene glycol) diacrylate
SDS	Sodium dodecyl sulfate
SEM	Scanning electron microscopy
SF	Silk fibroin
3D	Three-dimensional

## References

[1] Chartrain N.A., Gilchrist K.H., Ho V.B., Klarmann G.J. (2022). 3D bioprinting for the repair of articular cartilage and osteochondral tissue. Bioprinting.

[2] Lafuente-Merchan M., Ruiz-Alonso S., García-Villén F., Gallego I., Gálvez-Martín P., Saenz-del-Burgo L., Pedraz J.L. (2022). Progress in 3D Bioprinting Technology for Osteochondral Regeneration. Pharmaceutics.

[3] Vernengo A.J., Grad S., Eglin D., Alini M., Li Z. (2020). Bioprinting Tissue Analogues with Decellularized Extracellular Matrix Bioink for Regeneration and Tissue Models of Cartilage and Intervertebral Discs. Adv. Funct. Mater..

[4] Turnbull G., Clarke J., Picard F., Zhang W., Riches P., Li B., Shu W. (2020). 3D biofabrication for soft tissue and cartilage engineering. Med. Eng. Phys..

[5] Roseti L., Cavallo C., Desando G., Parisi V., Petretta M., Bartolotti I., Grigolo B. (2018). Three-Dimensional Bioprinting of Cartilage by the Use of Stem Cells: A Strategy to Improve Regeneration. Materials.

[6] Sahranavard M., Sarkari S., Safavi S., Ghorbani F. (2022). Three-dimensional bio-printing of decellularized extracellular matrix-based bio-inks for cartilage regeneration: A systematic review. Biomater. Transl..

[7] Mokhtarinia K., Masaeli E. (2023). Post-decellularized printing of cartilage extracellular matrix: Distinction between biomaterial ink and bioink. Biomater. Sci..

[8] Tan G., Xu J., Yu Q., Zhang J., Hu X., Sun C., Zhang H. (2022). Photo-Crosslinkable Hydrogels for 3D Bioprinting in the Repair of Osteochondral Defects: A Review of Present Applications and Future Perspectives. Micromachines.

[9] de Angelis E., Saleri R., Martelli P., Elviri L., Bianchera A., Bergonzi C., Pirola M., Romeo R., Andrani M., Cavalli V. (2021). Cultured Horse Articular Chondrocytes in 3D-Printed Chitosan Scaffold With Hyaluronic Acid and Platelet Lysate. Front. Vet. Sci..

[10] Kabirian F., Mozafari M. (2020). Decellularized ECM-derived bioinks: Prospects for the future. Methods.

[11] Ayariga J.A., Huang H., Dean D. (2022). Decellularized Avian Cartilage, a Promising Alternative for Human Cartilage Tissue Regeneration. Materials.

[12] Putra R.U., Basri H., Prakoso A.T., Chandra H., Ammarullah M.I., Akbar I., Syahrom A., Kamarul T. (2023). Level of Activity Changes Increases the Fatigue Life of the Porous Magnesium Scaffold, as Observed in Dynamic Immersion Tests, over Time. Sustainability.

[13] Park W., Gao G., Cho D.-W. (2021). Tissue-Specific Decellularized Extracellular Matrix Bioinks for Musculoskeletal Tissue Regeneration and Modeling Using 3D Bioprinting Technology. Int. J. Mol. Sci..

[14] Page M.J., McKenzie J.E., Bossuyt P.M., Boutron I., Hoffmann T.C., Mulrow C.D., Shamseer L., Tetzlaff J.M., Akl E.A., Brennan S.E. (2021). The PRISMA 2020 statement: An updated guideline for reporting systematic reviews. Syst. Rev..

[15] Goodarzi Hosseinabadi H., Dogan E., Miri A.K., Ionov L. (2022). Digital Light Processing Bioprinting Advances for Microtissue Models. ACS Biomater. Sci. Eng..

[16] Cheng C.W., Solorio L.D., Alsberg E. (2014). Decellularized tissue and cell-derived extracellular matrices as scaffolds for orthopaedic tissue engineering. Biotechnol. Adv..

[17] Escobar Ivirico J.L., Bhattacharjee M., Kuyinu E., Nair L.S., Laurencin C.T. (2017). Regenerative Engineering for Knee Osteoarthritis Treatment: Biomaterials and Cell-Based Technologies. Engineering.

[18] Toh W.S., Foldager C.B., Pei M., Hui J.H.P. (2014). Advances in Mesenchymal Stem Cell-based Strategies for Cartilage Repair and Regeneration. Stem Cell Rev. Rep..

[19] Zhang X., Liu Y., Luo C., Zhai C., Li Z., Zhang Y., Yuan T., Dong S., Zhang J., Fan W. (2021). Crosslinker-free silk/decellularized extracellular matrix porous bioink for 3D bioprinting-based cartilage tissue engineering. Mater. Sci. Eng. C.

[20] Cross M., Smith E., Hoy D., Nolte S., Ackerman I., Fransen M., Bridgett L., Williams S., Guillemin F., Hill C.L. (2014). The global burden of hip and knee osteoarthritis: Estimates from the Global Burden of Disease 2010 study. Ann. Rheum. Dis..

[21] Helmick C.G., Felson D., Lawrence R.C., Gabriel S., Hirsch R., Kwoh C.K., Liang M.H., Kremers H.M., Mayes M.D., Merkel P.A. (2007). Estimates of the prevalence of arthritis and other rheumatic conditions in the United States: Part I. Arthritis Rheum..

[22] Xu Y., Jia L., Wang Z., Jiang G., Zhou G., Chen W., Chen R. (2020). Injectable photo-crosslinking cartilage decellularized extracellular matrix for cartilage tissue regeneration. Mater. Lett..

[23] Hunziker E., Lippuner K., Keel M., Shintani N. (2015). An educational review of cartilage repair: Precepts & practice–myths & misconceptions–progress & prospects. Osteoarthr. Cartil..

[24] Commins J., Irwin R., Matuska A., Goodale M., Delco M., Fortier L. (2020). Biological Mechanisms for Cartilage Repair Using a BioCartilage Scaffold: Cellular Adhesion/Migration and Bioactive Proteins. Cartilage.

[25] Gupta P.K., Das A.K., Chullikana A., Majumdar A.S. (2012). Mesenchymal stem cells for cartilage repair in osteoarthritis. Stem Cell Res. Ther..

[26] Kim B.S., Das S., Jang J., Cho D.-W. (2020). Decellularized Extracellular Matrix-based Bioinks for Engineering Tissue- and Organ-specific Microenvironments. Chem. Rev..

[27] Sun W., Starly B., Daly A.C., Burdick J.A., Groll J., Skeldon G., Shu W., Sakai Y., Shinohara M., Nishikawa M. (2020). The bioprinting roadmap. Biofabrication.

[28] Goldberg A., Mitchell K., Soans J., Kim L., Zaidi R. (2017). The use of mesenchymal stem cells for cartilage repair and regeneration: A systematic review. J. Orthop. Surg. Res..

[29] Djouad F., Mrugala D., Noël D., Jorgensen C. (2006). Engineered mesenchymal stem cells for cartilage repair. Regen. Med..

[30] Gilpin A., Yang Y. (2017). Decellularization Strategies for Regenerative Medicine: From Processing Techniques to Applications. BioMed Res. Int..

[31] Qin C., Ma J., Chen L., Ma H., Zhuang H., Zhang M., Huan Z., Chang J., Ma N., Wu C. (2021). 3D bioprinting of multicellular scaffolds for osteochondral regeneration. Mater. Today.

[32] Kim H.S., Mandakhbayar N.-E., Kim H.-W., Leong K.W., Yoo H.S. (2020). Protein-reactive nanofibrils decorated with cartilage-derived decellularized extracellular matrix for osteochondral defects. Biomaterials.

[33] Levato R., Webb W.R., Otto I.A., Mensinga A., Zhang Y., van Rijen M., van Weeren R., Khan I.M., Malda J. (2017). The bio in the ink: Cartilage regeneration with bioprintable hydrogels and articular cartilage-derived progenitor cells. Acta Biomater..

[34] Kim Y.S., Majid M., Melchiorri A.J., Mikos A.G. (2018). Applications of decellularized extracellular matrix in bone and cartilage tissue engineering. Bioeng. Transl. Med..

[35] Zhang X., Chen X., Hong H., Hu R., Liu J., Liu C. (2021). Decellularized extracellular matrix scaffolds: Recent trends and emerging strategies in tissue engineering. Bioact. Mater..

[36] Galliger Z., Vogt C.D., Helms H.R., Panoskaltsis-Mortari A. (2022). Extracellular Matrix Microparticles Improve GelMA Bioink Resolution for 3D Bioprinting at Ambient Temperature. Macromol. Mater. Eng..

[37] Isaeva E.V., Beketov E.E., Demyashkin G.A., Yakovleva N.D., Arguchinskaya N.V., Kisel A.A., Lagoda T.S., Malakhov E.P., Smirnova A.N., Petriev V.M. (2022). Cartilage Formation In Vivo Using High Concentration Collagen-Based Bioink with MSC and Decellularized ECM Granules. Int. J. Mol. Sci..

[38] Setayeshmehr M., Hafeez S., van Blitterswijk C., Moroni L., Mota C., Baker M. (2021). Bioprinting Via a Dual-Gel Bioink Based on Poly(Vinyl Alcohol) and Solubilized Extracellular Matrix towards Cartilage Engineering. Int. J. Mol. Sci..

[39] Pati F., Jang J., Ha D.-H., Kim S.W., Rhie J.-W., Shim J.-H., Kim D.-H., Cho D.-W. (2014). Printing three-dimensional tissue analogues with decellularized extracellular matrix bioink. Nat. Commun..

[40] Xu H., Xu B., Yang Q., Li X., Ma X., Xia Q., Zhang Y., Zhang C., Wu Y., Zhang Y. (2014). Comparison of Decellularization Protocols for Preparing a Decellularized Porcine Annulus Fibrosus Scaffold. PLoS ONE.

[41] Elder B.D., Eleswarapu S.V., Athanasiou K.A. (2009). Extraction techniques for the decellularization of tissue engineered articular cartilage constructs. Biomaterials.

[42] Schwarz S., Koerber L., Elsaesser A.F., Goldberg-Bockhorn E., Seitz A.M., Dürselen L., Ignatius A., Walther P., Breiter R., Rotter N. (2012). Decellularized Cartilage Matrix as a Novel Biomatrix for Cartilage Tissue-Engineering Applications. Tissue Eng. Part A.

[43] Luo L., Eswaramoorthy R., Mulhall K.J., Kelly D.J. (2016). Decellularization of porcine articular cartilage explants and their subsequent repopulation with human chondroprogenitor cells. J. Mech. Behav. Biomed. Mater..

[44] Kiyotake E.A., Beck E.C., Detamore M.S. (2016). Cartilage extracellular matrix as a biomaterial for cartilage regeneration. Ann. N. Y. Acad. Sci..

[45] Peretti G.M., Randolph M.A., Caruso E.M., Rossetti F., Zaleske D.J. (1998). Bonding of cartilage matrices with cultured chondrocytes: An experimental model. J. Orthop. Res..

[46] Rowland C.R., Colucci L.A., Guilak F. (2016). Fabrication of anatomically-shaped cartilage constructs using decellularized cartilage-derived matrix scaffolds. Biomaterials.

[47] Vas W.J., Shah M., Blacker T.S., Duchen M., Sibbons P., Roberts S.J. (2018). Decellularized Cartilage Directs Chondrogenic Differentiation: Creation of a Fracture Callus Mimetic. Tissue Eng. Part A.

[48] Gong Y.Y., Xue J.X., Zhang W.J., Zhou G.D., Liu W., Cao Y. (2011). A sandwich model for engineering cartilage with acellular cartilage sheets and chondrocytes. Biomaterials.

[49] Stone R., Frahs S., Hardy M., Fujimoto A., Pu X., Keller-Peck C., Oxford J. (2021). Decellularized Porcine Cartilage Scaffold; Validation of Decellularization and Evaluation of Biomarkers of Chondrogenesis. Int. J. Mol. Sci..

[50] Hsueh M.-F., Khabut A., Kjellström S., Önnerfjord P., Kraus V.B. (2016). Elucidating the Molecular Composition of Cartilage by Proteomics. J. Proteome Res..

[51] Visscher D.O., Lee H., van Zuijlen P.P., Helder M.N., Atala A., Yoo J.J., Lee S.J. (2020). A photo-crosslinkable cartilage-derived extracellular matrix bioink for auricular cartilage tissue engineering. Acta Biomater..

[52] Groll J., Burdick J.A., Cho D.-W., Derby B., Gelinsky M., Heilshorn S.C., Jüngst T., Malda J., Mironov V.A., Nakayama K. (2019). A definition of bioinks and their distinction from biomaterial inks. Biofabrication.

[53] Beck E.C., Barragan M., Tadros M.H., Gehrke S.H., Detamore M.S. (2016). Approaching the compressive modulus of articular cartilage with a decellularized cartilage-based hydrogel. Acta Biomater..

[54] Han Y., Lian M., Zhang C., Jia B., Wu Q., Sun B., Qiao Z., Sun B., Dai K. (2022). Study on bioactive PEGDA/ECM hybrid bi-layered hydrogel scaffolds fabricated by electro-writing for cartilage regeneration. Appl. Mater. Today.

[55] Wu J., Han Y., Fu Q., Hong Y., Li L., Cao J., Li H., Liu Y., Chen Y., Zhu J. (2022). Application of tissue-derived bioink for articular cartilage lesion repair. Chem. Eng. J..

[56] Behan K., Dufour A., Garcia O., Kelly D. (2022). Methacrylated Cartilage ECM-Based Hydrogels as Injectables and Bioinks for Cartilage Tissue Engineering. Biomolecules.

[57] Herrera Millar V.R., Canciani B., Mangiavini L., Filipe J.F.S., Aidos L., Pallaoro M., Peretti G.M., Pocar P., Modina S.C., Di Giancamillo A. (2022). Endostatin in 3D Fibrin Hydrogel Scaffolds Promotes Chondrogenic Differentiation in Swine Neonatal Meniscal Cells. Biomedicines.

[58] Eichholz K.F., Freeman F.E., Pitacco P., Nulty J., Ahern D., Burdis R., Browe D.C., Garcia O., Hoey D.A., Kelly D.J. (2022). Scaffold microarchitecture regulates angiogenesis and the regeneration of large bone defects. Biofabrication.

[59] Terpstra M.L., Li J., Mensinga A., de Ruijter M., van Rijen M.H.P., Androulidakis C., Galiotis C., Papantoniou I., Matsusaki M., Malda J. (2022). Bioink with cartilage-derived extracellular matrix microfibers enables spatial control of vascular capillary formation in bioprinted constructs. Biofabrication.

[60] Yuan Z., Lyu Z., Liu X., Zhang J., Wang Y. (2021). Mg-BGNs/DCECM Composite Scaffold for Cartilage Regeneration: A Preliminary In Vitro Study. Pharmaceutics.

[61] Bedell M.L., Torres A.L., Hogan K.J., Wang Z., Wang B., Melchiorri A.J., Grande-Allen K.J., Mikos A.G. (2022). Human gelatin-based composite hydrogels for osteochondral tissue engineering and their adaptation into bioinks for extrusion, inkjet, and digital light processing bioprinting. Biofabrication.

[62] Rathan S., Dejob L., Schipani R., Haffner B., Möbius M.E., Kelly D.J. (2019). Fiber Reinforced Cartilage ECM Functionalized Bioinks for Functional Cartilage Tissue Engineering. Adv. Healthc. Mater..

[63] Chen W., Xu Y., Li Y., Jia L., Mo X., Jiang G., Zhou G. (2019). 3D printing electrospinning fiber-reinforced decellularized extracellular matrix for cartilage regeneration. Chem. Eng. J..

[64] Prakoso A.T., Basri H., Adanta D., Yani I., Ammarullah M.I., Akbar I., Ghazali F.A., Syahrom A., Kamarul T. (2023). The Effect of Tortuosity on Permeability of Porous Scaffold. Biomedicines.

